# A Dual-Branch CNN-Based Method for Satellite Navigation Jamming Classification and Parameter Estimation

**DOI:** 10.3390/s26165251

**Published:** 2026-08-19

**Authors:** Teng Zhao, Yongqing Wang, Lixun Li, Yanbo Luo, Chenhao Zhao, Lixin Zhang

**Affiliations:** 1 School of Information and Navigation, Air Force Engineering University, Xi’an 710077, China; 18173667658@163.com (T.Z.); lilixun1985@163.com (L.L.); lybob0515@163.com (Y.L.); 14291024@bjtu.edu.cn (C.Z.); 2 Xi’an Institute of Space Radio Technology, Xi’an 710061, China; 13991927780@163.com

**Keywords:** BeiDou-3 Satellite Navigation System (BDS-3), suppressive jamming, blind suppression, precision suppression, jamming classification, parameter estimation

## Abstract

The BeiDou-3 Satellite Navigation System (BDS-3) serves as a critical national space-based information infrastructure. However, the signals received at user terminals are characterized by power levels that lie significantly below the noise floor, rendering them highly vulnerable to both intentional suppressive jamming and unintentional interference in complex electromagnetic environments. Traditional anti-jamming techniques predominantly rely on blind suppression strategies. While effective against simple jamming scenarios, these methods suffer from inherent limitations, including low resource utilization and poor adaptability to complex, time-varying interference. To achieve precise suppression and real-time detection of interference, it is necessary to simultaneously perform classification and parameter estimation of the jamming signal. To this end, this paper proposes a multi-task dual-branch convolutional neural network (CNN) learning framework that encompasses four typical types of suppressive jamming: continuous wave interference (CWI), wideband noise interference (WNI), pulse interference (PI), and chirp interference (CI). Taking time-frequency spectrograms of signals as input, the framework achieves accurate identification of interference types and simultaneous estimation of key physical parameters. Simulation and experimental results demonstrate that the proposed method maintains stability and feasibility under low-INR conditions.

## 1. Introduction

The BeiDou-3 Satellite Navigation System (BDS-3) has been extensively deployed across critical sectors such as transportation, power grid communications, and national defense, establishing itself as a fundamental component of societal operations and navigation warfare systems. The realization of its high-precision positioning and timing services fundamentally relies on the reliable reception of downlink satellite signals by user terminals. However, after traversing vast distances through space, these navigation signals arrive at the Earth’s surface with extremely weak power, often falling below the levels of environmental noise and various interfering signals. This inherent vulnerability makes the system highly susceptible to Radio Frequency Interference (RFI), which can lead to degraded positioning accuracy, loss of signal lock, or even complete denial of navigation capability [1,2,3,4].

To enhance the performance of satellite navigation systems in complex electromagnetic environments, blind suppression techniques have been developed as critical countermeasures. Their core design principle is to mitigate interference without relying on prior knowledge of the jamming type or parameters, utilizing methods such as time-domain adaptive filtering, frequency-domain broadband notch filtering, and space–time adaptive processing (STAP). However, the lack of specificity in these approaches leads to inherent limitations. These limitations have remained fundamentally unresolved after decades of development: time-domain adaptive filters often exhibit insufficient dynamic tracking capability and are prone to divergence under strong interference [5]; frequency-domain notch filtering is constrained by the time-domain truncation effect, making complete interference cancellation difficult [6,7]; blind spatial filtering can introduce carrier phase offsets [8]; and blind STAP may cause signal distortion and measurement errors [9].

To overcome the deficiencies of blind suppression, achieving targeted suppression is paramount. The implementation of targeted suppression primarily depends on the accurate classification of interference signals. Various methods have been applied in this regard. For instance, Li Fangyuan et al. [10] proposed a method that combines Short-Time Fourier Transform (STFT) for time-frequency feature extraction with a Support Vector Machine (SVM) classifier to effectively identify three types of active jamming signals in radar systems. Xu Hongkui et al. [11] established a classification model based on a Weighted Random Forest for uplink interference types in FDD-LTE systems, improving the identification accuracy for minority-class interference through class weight assignment. He Z. et al. [12] proposed a method for identifying communication signal modulation patterns based on a Bayesian optimization-based decision tree, which is capable of recognizing three signal types—Frequency Shift Keying (FSK), Frequency Modulation (FM), and Amplitude Modulation (AM)—with high accuracy. Second, following accurate classification, precise estimation of jamming parameters is required to effectively integrate with anti-jamming techniques. Similarly, multiple methods exist for parameter estimation. Ranney K et al. [13] investigated a non-coherent pulse width estimation algorithm based on Haar filters, demonstrating high detection rates and low error under low Signal-to-Noise Ratio (SNR) conditions in experiments. Sun Zhiguo et al. [14] proposed a method for Linear Frequency Modulated (LFM) signal parameter estimation based on a Two-Stage Local Polynomial Fourier Transform (TSLPFT) and FFT, estimating the chirp rate via a two-stage search. Their approach subsequently estimating the center frequency and sweep rate using FFT. Astfalck L C et al. [15] introduced a debiased Welch power spectrum estimation method, which fits the Welch estimates using a constructed set of biased basis functions and then recovers the unbiased power spectral density via weighted least squares. This approach significantly reducing estimation bias from finite samples while maintaining computational efficiency. However, these traditional classification and parameter estimation methods predominantly rely on handcrafted features, and exhibit poor adaptability to non-stationary interference.

With the rapid advancement of artificial intelligence, deep learning has successfully overcome the reliance on expert knowledge inherent in traditional methods by leveraging its powerful capabilities for automatic feature extraction, complex pattern recognition, and nonlinear fitting. This approach has demonstrated unique advantages in processing high-dimensional, non-stationary, and noisy signal data. Mehr I E et al. [16] proposed a feature-aided Convolutional Neural Network (CNN) for GNSS interference classification, achieving high accuracy even under low-power jamming by combining time-frequency images with statistical features. Morales et al. [17] employed black-and-white time-frequency spectrograms as input to classify multiple types of interference signals using a convolutional neural network and a support vector machine, achieving high classification accuracy. Wang P Y et al. [18] proposed an interference recognition algorithm based on a convolutional neural network combined with multi-domain feature extraction (CNN-JMDFE). By simultaneously extracting interference features automatically from time-frequency images and frequency-domain sequences, the method significantly improves the classification accuracy for both single and composite interference in wireless communication systems under low jam-to-noise ratio conditions. Yan B M et al. [19] addressed the complex challenge of parameter estimation for multi-component linear frequency modulation (LFM) signals by introducing an innovative convolutional neural network-based high-resolution Fractional Fourier Transform (FrFT) parameter estimation approach. Yan K et al. [20] proposed a Deep Adaptive Temporal Network (DAT-Net) for the high-precision numerical estimation of specific parameters (e.g., bandwidth, pulse width, number of sub-pulses) of radar multipath interference signals. Their method demonstrates superior performance in both single-parameter and multi-parameter estimation tasks. The aforementioned research indicates that neural network-based approaches for combined interference classification and parameter estimation significantly outperform traditional methods in handling high-dimensional complex interference, adapting to environmental changes, and enabling end-to-end optimization, thereby enhancing the efficacy of targeted suppression. Nonetheless, these methods typically adopt a sequential “classify-then-estimate” architecture, which can lead to fragmented feature representation and cumulative processing delays, resulting in poor real-time performance and resource redundancy.

Taking this as a starting point, this paper designs a dual-branch CNN-based method for jamming classification and parameter estimation, using time-frequency spectrograms of four types of intentional suppressive jamming—CWI, WNI, PI, and CI—and the no-jam scenario as input. The method performs feature extraction via a shared backbone network and jointly trains parallel classification and regression branches under a multi-task learning framework, thereby simultaneously achieving signal identification and parameter estimation in a single forward pass. This effectively resolves the issues of fragmented feature representation and delay accumulation inherent in serial architectures, significantly improving the real-time performance of jamming signal processing and reducing resource occupancy.

## 2. Input Signal Modeling and Time-Frequency Feature Extraction

**Figure 1:**
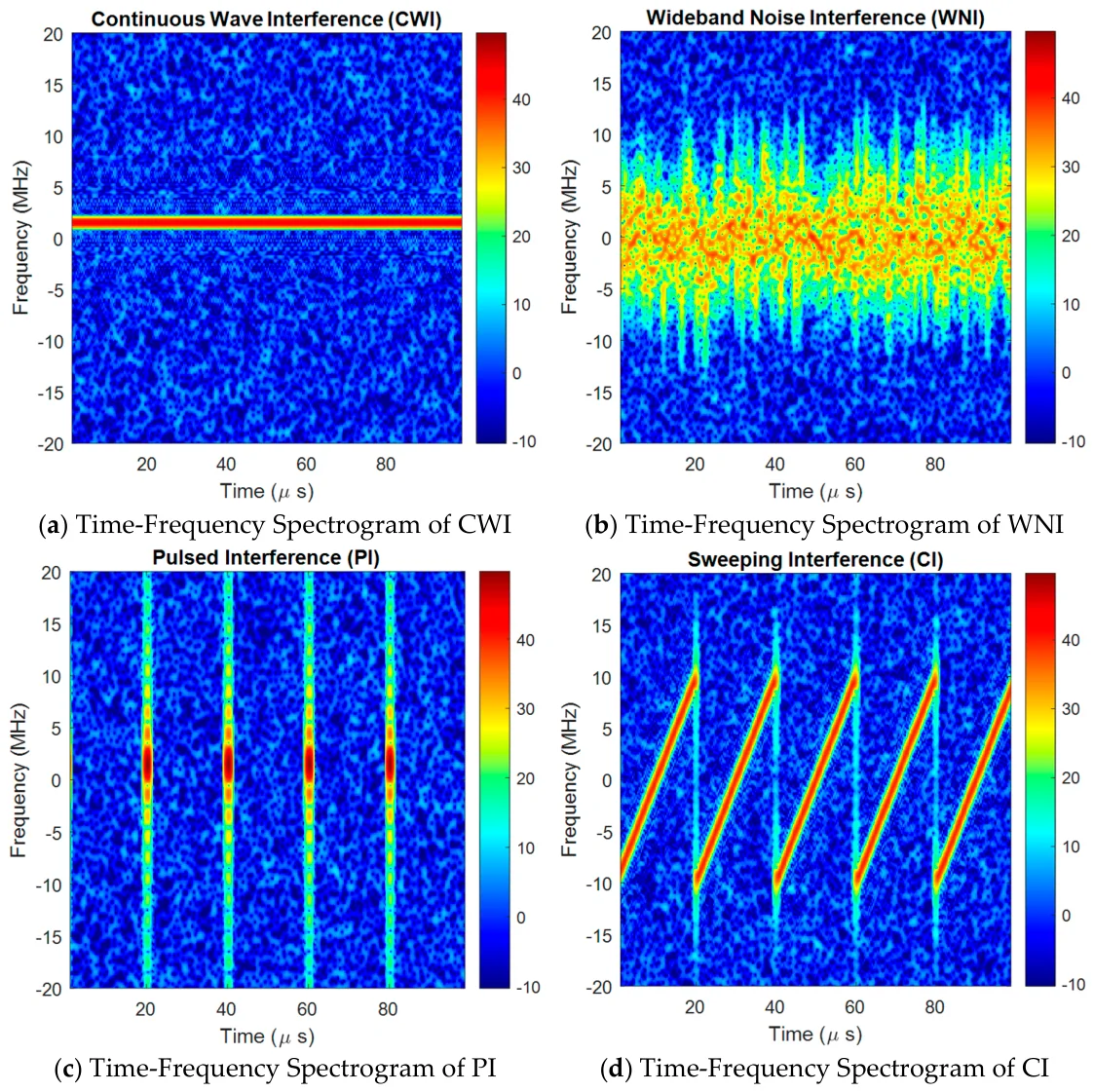
Time-frequency diagrams of interference signals.

This section focuses on four typical types of intentional suppressive jamming: CWI, WNI, PI, and CI. It establishes their mathematical models in complex baseband signal form and describes the signal preprocessing and time-frequency analysis procedures. Figure 1 illustrates the time-frequency characteristics of these jamming types.

### 2.1. Mathematical Modeling of Signals

#### 2.1.1. Continuous Wave Interference (CWI)

Continuous Wave Interference represents the simplest form of suppressive jamming. It operates by transmitting a sinusoidal wave at a fixed frequency to occupy a specific narrow band, thereby overpowering the satellite navigation signal within that band. Its mathematical model is given by:

(1) $$s_{CWI}=A\cdot exp\{j(2\pi f_{c}t+\varphi_{0})\},$$

where A is the interference amplitude, fc is the center frequency, and φ0 is the initial phase.

#### 2.1.2. Wideband Noise Interference (WNI)

Broadband Noise Interference is configured as a noise-modulated frequency modulation (NMFM) signal, which manifests as continuous, irregular frequency fluctuations on the time-frequency diagram. By dynamically modulating frequency, it simultaneously degrades carrier tracking accuracy and correlator peak detection, making it difficult for satellite navigation receivers to acquire and track weak signals. Its mathematical model is:

(2) $$s_{WNI}(t)=Acos\left[2\pi f_{c}t+2\pi k_{FM}\int_{0}^{t}n(\tau )d\tau +\varphi_{0}\right],$$

where A is the interference amplitude, $f_{c}$ is the carrier frequency, $\varphi_{0}$ is the initial phase, $k_{FM}$ is the frequency modulation sensitivity, and n(t) is the normalized low-frequency modulating noise, defined as

(3) $$n(t)=\frac{w(t)*h_{LPF}(t)}{max(\left|w(t)*h_{LPF}(t)\right|)},$$

with w(t)~N(0,1) and $h_{LPF}(t)$ being a low-pass filter with bandwidth B.

#### 2.1.3. Pulsed Interference (PI)

Pulsed interference emits high-power signals in short pulses, instantly saturating the receiver front-end, constituting a peak-suppression type of jamming. Its mathematical model references the characteristics of DME signals:

(4) $$s_{PI}(t)=A\cdot \sum_{k=1}^{K}\left(p(t-kT_{p})\cdot exp\{j(2\pi f_{c}t+\varphi_{0})\}\right),$$

where A is the pulse peak amplitude, $T_{p}$ is the pulse repetition period, $f_{c}$ is the carrier frequency, $\varphi_{0}$ is the initial phase, and p(t) is the Gaussian pulse model given by

(5) $$p(t)=exp(-\frac{t^{2}}{2\sigma_{p}^{2}}).$$

Here, $\sigma_{p}$ determines the pulse width $\tau_{p}$ (typically $\tau_{p}\approx 2.3548\sigma_{p}$).

#### 2.1.4. Chirp Interference (CI)

Chirp interference features frequency that varies linearly or non-linearly over time, covering a broad frequency band to impose wideband dynamic suppression on satellite navigation signals. This study focuses on the most common linear chirp. Its mathematical model is:

(6) $$s_{CI}(t)=A\cdot exp\{j(2\pi \int_{0}^{t}f(\tau )d\tau +\varphi_{0})\},$$

where $f\left(\tau \right)$ = $f_{0}\;$+ k·t. Here, A is the interference amplitude, $f_{0}$ is the starting frequency, $\varphi_{0}$ is the initial phase, and k is the chirp rate.

This research focuses on the four types of interference described above, which collectively cover the primary categories of interference signal time-frequency structures. Therefore, this set can be considered a quasi-complete feature set capable of effectively testing the ability of intelligent perception models to handle challenges posed by different characteristics.

### 2.2. Signal Preprocessing and Time-Frequency Analysis

#### 2.2.1. Signal Preprocessing

**Figure 2:**
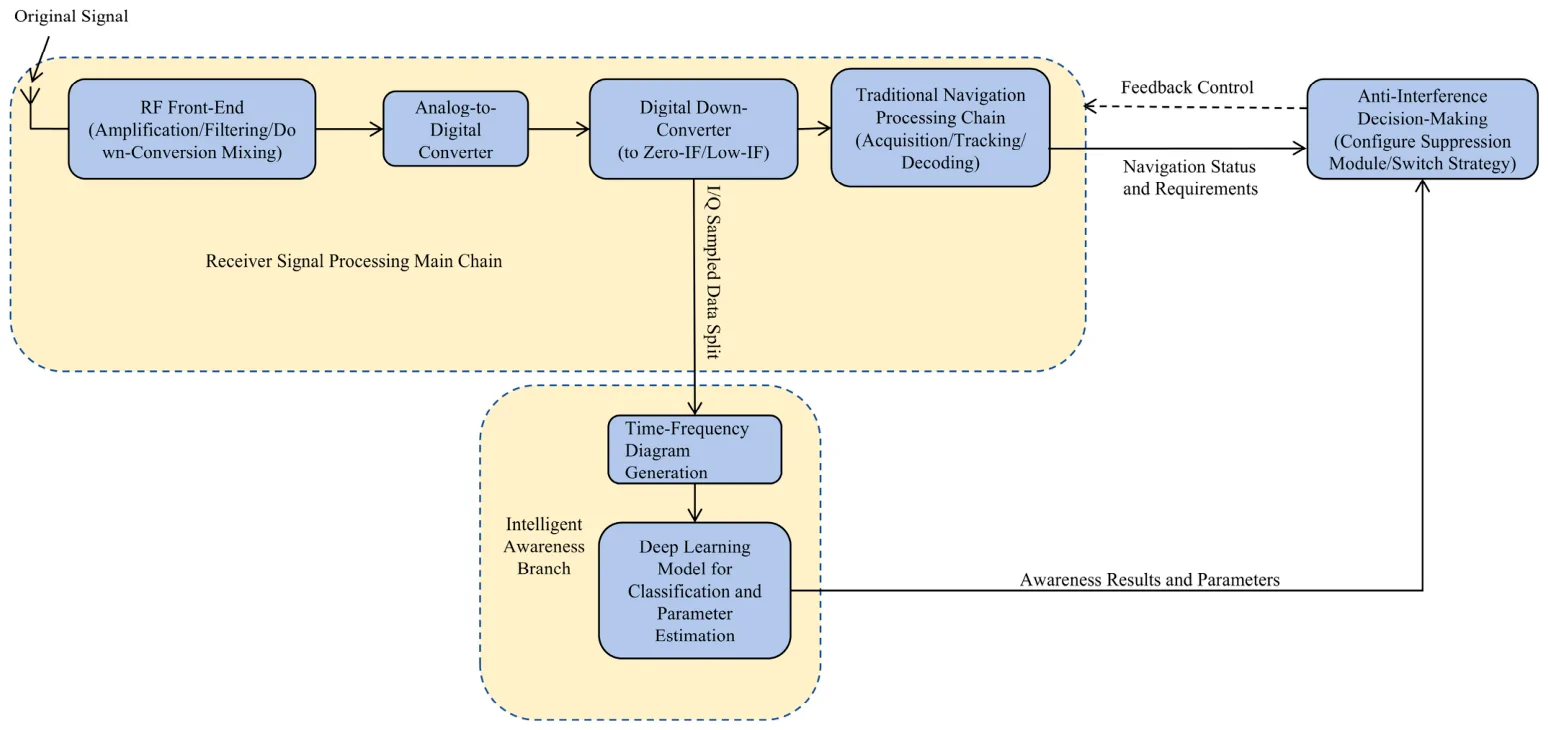
Signal processing flowchart.

Upon entering the receiver, navigation signals cannot be directly subjected to time-frequency analysis. This limitation stems from the inherent physical characteristics of the signals and the practical constraints of engineering implementation. The signal processing flow is illustrated in Figure 2.

As shown in Figure 2, the original navigation signal arrives at the receiver antenna in the form of high-frequency electromagnetic waves and must undergo a series of preprocessing steps before time-frequency analysis can be performed. First, the signal passes through the receiver RF front-end, where it sequentially undergoes low-noise amplification (to boost the weak signal level), RF filtering (to suppress out-of-band interference and image frequencies), and down-conversion (by mixing with a local oscillator signal to translate the RF signal to an intermediate frequency). This significantly reduces the signal’s center frequency, facilitating subsequent digital processing. The analog IF signal is then fed into an analog-to-digital converter for bandpass sampling, outputting a digital IF signal—at this point, the signal has been converted from the analog to the digital domain and is preliminarily amenable to computation. However, since the digital IF signal still contains a relatively high residual carrier and a large data volume, it must be further processed by digital down-conversion (DDC). Through digital mixing and decimation filtering, the IF signal is translated to baseband while the sampling rate is reduced, ultimately yielding a zero-IF baseband I/Q quadrature signal.

Throughout the entire preprocessing chain, the generation of the baseband I/Q signal serves as a critical node linking front-end reception with back-end intelligent processing. Its significance is threefold: First, the zero-IF baseband signal preserves the complete amplitude and phase information of the interference signal, providing a high-fidelity data source for the STFT spectrogram; second, compared with RF or IF signals, the baseband I/Q signal features a lower data rate and reduced storage requirements, meeting the timeliness demands of subsequent batch processing; third, the baseband I/Q signal has not yet entered the conventional navigation processing chain (acquisition, tracking, demodulation, etc.), meaning the energy characteristics of the interference remain unaltered or undiluted by internal receiver processing, thus most faithfully reflecting the original time-frequency structure of the interference. It can be seen, therefore, that the essential function of this signal preprocessing flow is to accomplish the transformation from physically propagated signals in space to standardized feature maps suitable for deep learning model input, providing information-complete and quality-controllable input for the proposed dual-branch CNN, which constitutes the logical starting point of the entire closed loop from perception to estimation to countermeasure.

#### 2.2.2. Principle of Time-Frequency Analysis

To balance computational complexity against representational capacity and to generate time-frequency representations suitable for Convolutional Neural Network (CNN) processing, this study employs the Short-Time Fourier Transform (STFT) for signal time-frequency analysis. Its core principle involves segmenting the signal into a series of short-term quasi-stationary fragments using a finite-length window function that slides along the time axis, followed by a Fourier Transform performed on each fragment. This yields the local spectrum, revealing the evolution of the signal’s frequency components over time.

For a discrete complex baseband signal X(n), the mathematical definition of its STFT is:

(7) $$X(m,k)=\sum_{n=0}^{L-1}x[n+m]\cdot w[n]\cdot e^{-j\frac{2\pi }{N}kn}.$$

The physical meanings of the terms are as follows:

X(n): The discrete-time complex baseband signal sequence, resulting from ADC sampling and subsequent down-conversion, containing amplitude and phase information along the time dimension.

w(n): A window function of length L (e.g., Hamming or Hanning window). Its role is to localize the analysis in the time domain, focusing only on the signal segment within the current window, thereby achieving localization from the global signal to specific time intervals.

m: The time frame index, which determines the position of the window function along the time axis.

k: The frequency index, corresponding to the digital angular frequency $w_{k}=\frac{2\pi }{N}k$. The index k constructs the frequency dimension of the time-frequency plot. N is the number of FFT points.

The time-frequency plot (spectrogram) input to the neural network is a two-dimensional visual representation of the STFT results. The horizontal axis represents time information, and the vertical axis represents frequency information. The color or grayscale value represents the energy magnitude of the corresponding time-frequency unit:

(8) $$S(m,k)=10\cdot {log}_{10}({\left|X(m,k)\right|}^{2}+\epsilon )[dB],$$

where ϵ is a minimal positive constant to prevent arithmetic underflow. In a color spectrogram, warmer colors indicate higher energy at a given time-frequency point, while in a grayscale spectrogram, a higher grayscale value indicates higher energy.

## 3. Design of the Dual-Branch CNN-Based Model Network

**Figure 3:**
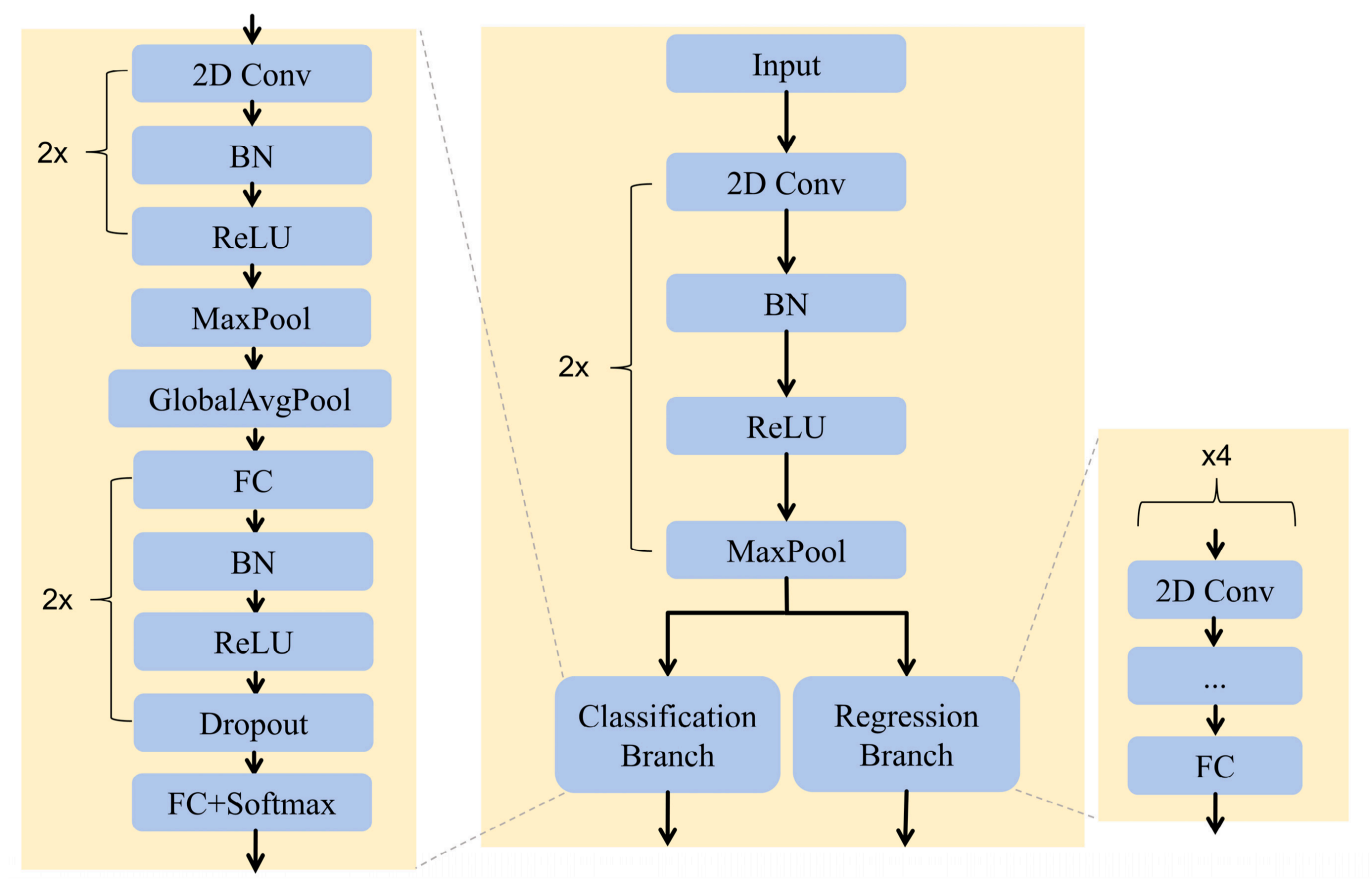
Model architecture.

The dual-branch CNN-based model takes the short-time Fourier transform (STFT) time-frequency spectrograms of jamming signals as input, extracts fundamental time-frequency features through a shared shallow convolutional network, and then diverges into two independent deep task branches to perform jamming classification and parameter regression, respectively. A schematic diagram of the model architecture is shown in Figure 3.

1. Input Laye: receives five standardized time-frequency spectrograms as unified input, corresponding to four types of interference—CWI, WNI, PI, and CI—and the no-jam scenario. The spectrograms are generated via short-time Fourier transform and encapsulate the complete time-frequency characteristics of the jamming signals. It should be noted that the premise of treating a single interference source as the processing target lies in the fact that the system’s front-end framework decouples composite interference into several independent components through array direction finding and spatial filtering, thereby rendering single-source extraction a feasible and reasonable basic unit. Consequently, the network input circumvents the identification ambiguity and estimation bias caused by multi-component coupling and reduces the complexity of directly modeling composite signals in multi-task learning. The entire interference processing chain regards the front-end decoupling of composite interference and the back-end type identification/parameter estimation as two independent stages—the former being accomplished by array direction finding and spatial filtering, while the latter, which is the focus of this study, performs refined perception based on the single-source components output by the former. This layered decoupling ensures that each stage of the interference processing chain can be optimized independently, and also justifies the research focus of this paper on the core issue of precise perception after decoupling as a reasonable investigation setting.
2. Shared feature extraction layer: The shared feature extraction module consists of two consecutive convolutional blocks. Each block comprises a 2D convolutional layer (Conv2D), batch normalization, a ReLU activation function, and max pooling. The first convolutional layer has a kernel size of 3 × 3 with 32 channels and a stride of 1; the second convolutional layer also has a kernel size of 3 × 3, with the channel number expanded to 64. This module is designed to extract general time-frequency features from the input spectrograms, providing a shared foundational feature representation for the subsequent task branches.
3. Independent branch layer: First, the regression targets of the branching layers are defined. These parameters determine the configuration logic of the corresponding anti-interference measures and form the basis for achieving precise suppression. The relevant contents are shown in Table 1. The four parameters selected for the regression tasks cover, in a physical sense, three fundamental dimensions: frequency-domain location (CWI center frequency), frequency-domain range (CI start and stop frequencies), and time-domain period (CI sweep period). Characteristics such as the bandwidth of WNI and the pulse width of PI can be indirectly characterized by the above parameter dimensions; therefore, independent regression branches are not set for them at this stage. The independent branch layer performs feature distribution by simultaneously replicating the features output from the shared feature extraction layer and feeding them into five independent branches, comprising one classification branch and four regression branches. Each branch shares an identical two-layer convolutional structure but maintains independent parameters, ensuring the task specificity of the high-level feature representations. The specific structure of the branches is as follows: First, two sequential 2D convolutional + batch normalization + ReLU modules are employed to extract deep spatial features from the image, and max pooling is used for down-sampling to reduce the spatial resolution. Subsequently, global average pooling compresses the feature maps into a one-dimensional feature vector, which preserves the global semantic information while significantly reducing the number of parameters in the fully connected layers. This vector is then passed through two structurally identical fully connected blocks (each containing a fully connected layer, batch normalization, ReLU activation, and Dropout regularization) for high-level feature integration and overfitting mitigation. Finally, an output fully connected layer generates the final prediction result (with an additional softmax activation in the classification branch to convert the output into a probability distribution).
4. Loss function: The total loss is the weighted sum of the classification cross-entropy loss and the parameter estimation error loss:
  (9) $$L_{MTL}=\alpha_{c}L_{cls}+\sum_{j=1}^{4}\alpha_{r_{j}}L_{{reg}_{j}},$$
  where $\alpha_{c}$ denotes the classification weight, $\alpha_{r_{j}}$ denotes the four regression weights, $L_{cls}$ is the cross-entropy loss, and $L_{reg}^{\left(j\right)}$ denotes the four error losses.
  (10) $$L_{cls}=-\frac{1}{N}\sum_{i=1}^{N}\sum_{j=1}^{5}y_{cls,i}^{\left(j\right)}ln{\hat{y}}_{cls,i}^{\left(j\right)}$$
  (11) $$L_{reg}^{\left(j\right)}=\frac{1}{N}\sum_{i=1}^{N}{\left({\hat{y}}_{reg,i}^{\left(j\right)}-y_{reg,i}^{\left(j\right)}\right)}^{2}$$
  Here, N denotes the number of samples; $y_{cls,i}^{\left(j\right)}$ is the ground-truth label value of the j-th class for the i-th sample; ${\hat{y}}_{cls,i}^{\left(j\right)}$ is the estimated label probability for the i-th sample and j-th class; ${\hat{y}}_{reg,i}^{\left(j\right)}$ is the estimated value of the j-th parameter for the i-th sample; and $y_{reg,i}^{\left(j\right)}$ is the corresponding ground-truth parameter value.
  Based on dimensional analysis, the loss weights are set to 1.0 for the classification loss, the center frequency, and the chirp start/end frequencies, while the weight for the chirp sweep period loss is set to 2.2.
5. Output layer: Outputs the classification and parameter estimation results of the model.

It should be noted that a relatively simple CNN architecture was adopted in this study. The rationale is threefold: first, the research objective is not to propose a novel backbone network for pursuing state-of-the-art accuracy, but rather to investigate the mechanism by which the sharing depth in a hard parameter sharing architecture affects multi-task performance—which requires that experiments be conducted under the principle of controlled variables. Employing a simple CNN as the base architecture ensures that the performance differences observed in the ablation experiments can be primarily attributed to variations in the number of shared layers, rather than to other confounding factors. Second, the physical nature of spectrogram inputs naturally matches the local receptive fields of convolutional operations, allowing a simple CNN to effectively extract their time-frequency features. Third, the resource constraints (real-time performance and lightweight design) of the target deployment platform further limit the feasibility of more complex models.

## 4. Analysis of Simulation Results

### 4.1. Dataset Construction and Experimental Procedure Analysis

To evaluate the performance of the algorithm in near-real electromagnetic environments, data generation was carried out using the navigation signal simulation system in the laboratory. Operating under a common time base, the system can synchronously output BeiDou satellite signals, suppressive jamming signals, and background noise, all with precisely adjustable parameters. After these signals are composited at the RF stage via a combiner, their physical characteristics closely approximate those of a real receiving environment. The specific configuration parameters are provided in Table 2. To construct the training input, the composite signals undergo analog-to-digital conversion and down-conversion, are collected as baseband I/Q sample files, and are then transformed into time-frequency spectrograms via short-time Fourier transform in MATLAB R2023a.

To evaluate the performance of the algorithm under different interference strengths, the dataset was constructed within an INR range of −10 dB to 30 dB. The training set and Test Sets 1 and 3 were sampled uniformly and randomly within this range. A total of 10,000 samples were generated for the training set, covering five scenarios (no-jam and the four types of interference), with an equal number of samples allocated to each scenario. Test Set 1, comprising a total of 10,000 samples, was used for overall performance evaluation. Test Set 2 was generated at five discrete INR levels (−10, 0, 10, 20, and 30 dB), with 5,000 samples per level, for refined performance comparison under different INR conditions. Test Set 3, built upon Test Sets 1 and 2, incorporated multipath effects and Doppler shifts with random system parameters through the navigation simulation system, generating a total of 10,000 samples as a challenging test set. The samples of each interference type were also evenly distributed across all three test sets. Meanwhile, the regression parameters for the CWI and CI samples were uniformly and randomly sampled within their specified ranges.

The training environment configuration was as follows: Windows 11 operating system, CPU: AMD Ryzen 7 5800H, GPU: NVIDIA GeForce RTX 3060, and 16 GB of RAM. During training, the Adam optimizer was adopted with an initial learning rate of 0.001. The batch size was set to 64, and training was conducted for a total of 120 epochs.

**Figure 4:**
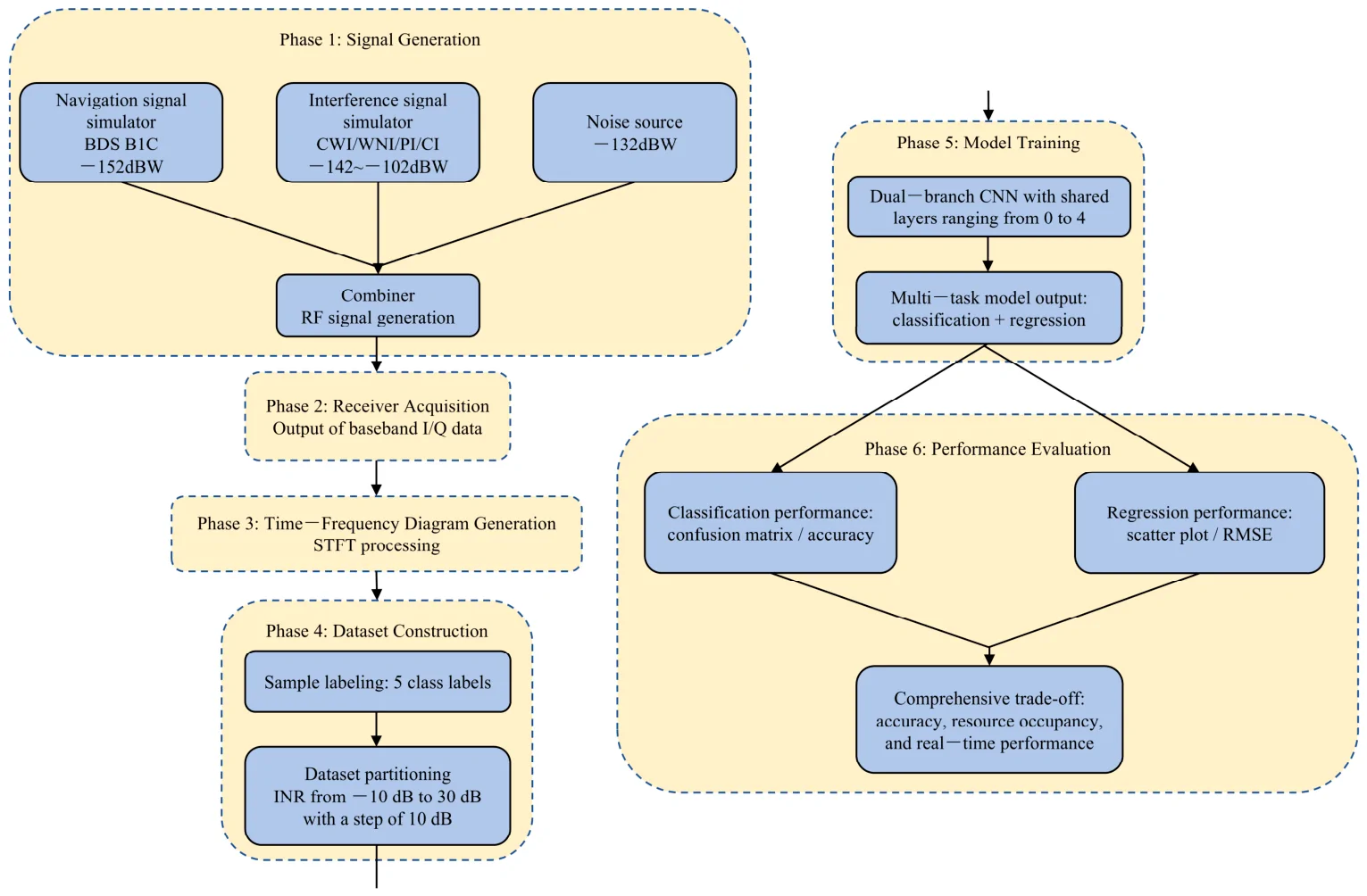
Flowchart of simulation experiment data generation, model training, and evaluation.

Figure 4 illustrates the complete experimental workflow from data generation to model evaluation under the laboratory simulation environment. The entire process is divided into six stages: (1) Simulated signal generation and RF combining: under a unified time base, the navigation signal simulator, interference signal simulator, and noise source output synchronously to generate the BeiDou B1C signal, four typical types of suppressive interference, and background noise, respectively, with independently adjustable parameters. The three signal paths are combined via a combiner into an RF analog signal. (2) Receiver acquisition: the RF analog signal is acquired by the receiver to obtain baseband I/Q data, with specific details shown in Figure 2. (3) Spectrogram construction: the baseband I/Q data are framed and processed via STFT to generate time-frequency spectrograms, followed by normalization. (4) Dataset construction: the training set and test sets are partitioned, with INR ranging from −10 dB to 30 dB and balanced distribution across all interference types. (5) Model training: models with 0 to 4 shared layers are trained to output classification and regression results. (6) Performance evaluation: on the test sets, the classification confusion matrix, classification accuracy, parameter estimation scatter plots, and RMSE metrics are produced.

It should be noted that this figure focuses on presenting the complete experimental workflow from data generation to model evaluation, while the specific signal processing details of receiver acquisition and spectrogram generation are already elaborated in Figure 2. The two figures present complementary perspectives—one from the experimental workflow and the other from the actual signal processing—rather than being redundant.

### 4.2. Analysis of Experimental Results

#### 4.2.1. Error Analysis

**Figure 5:**
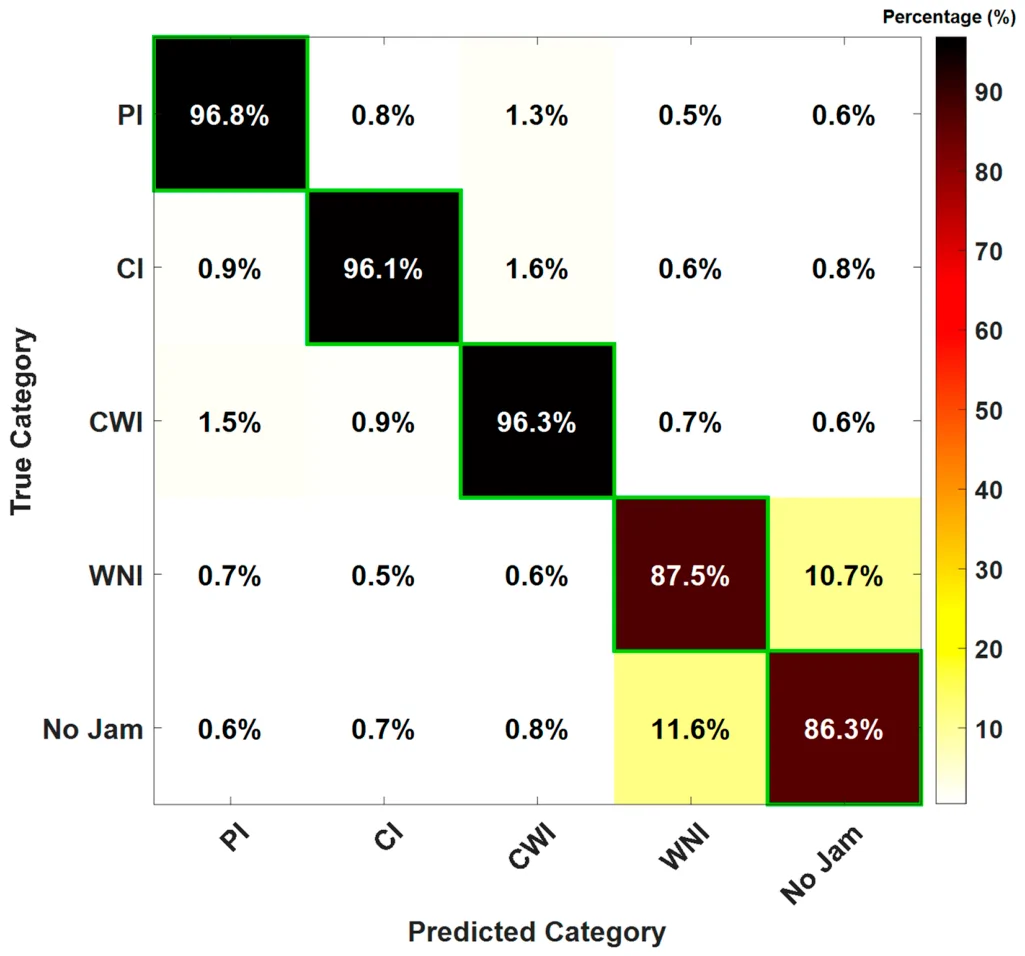
Confusion matrix.

**Figure 6:**
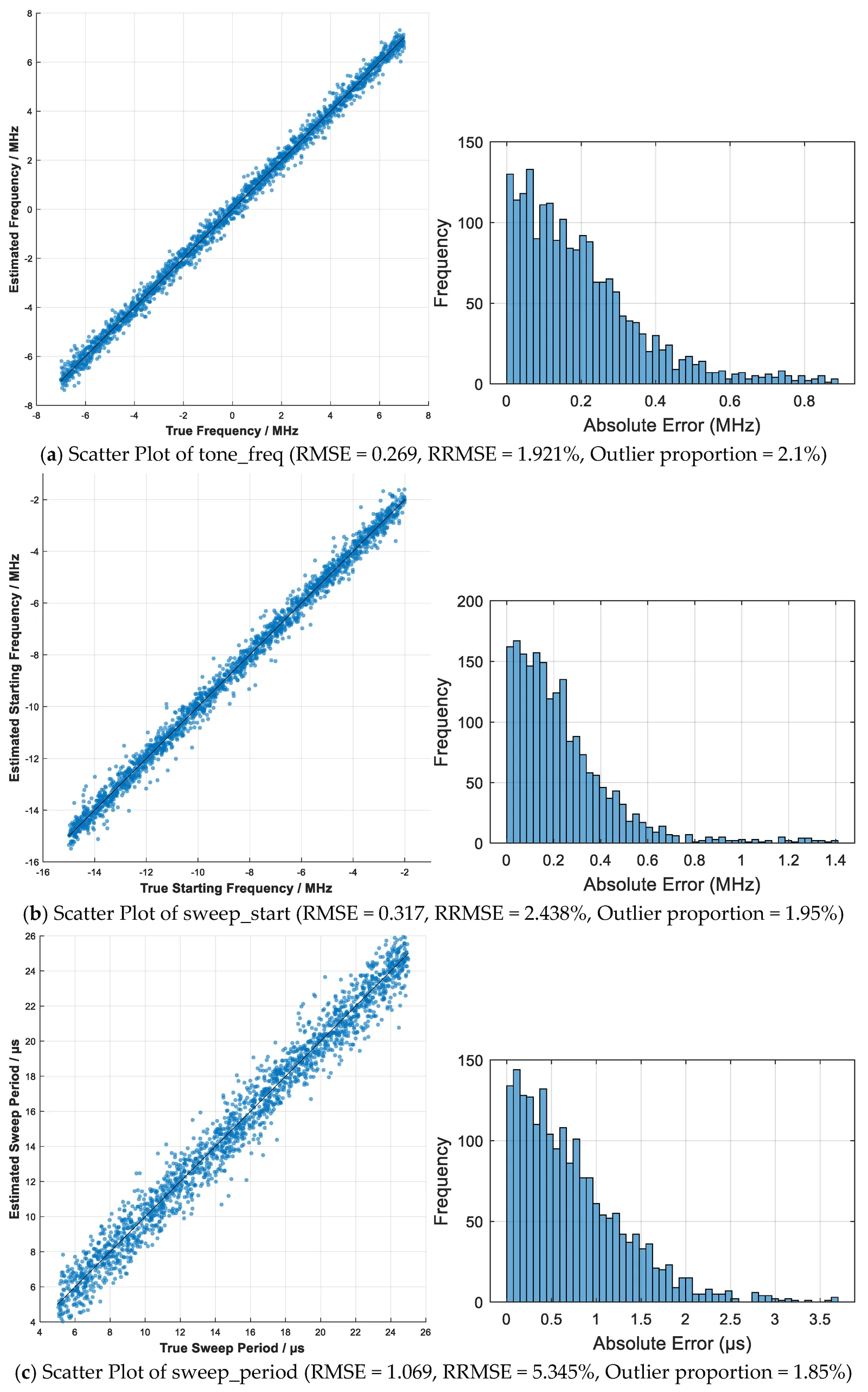

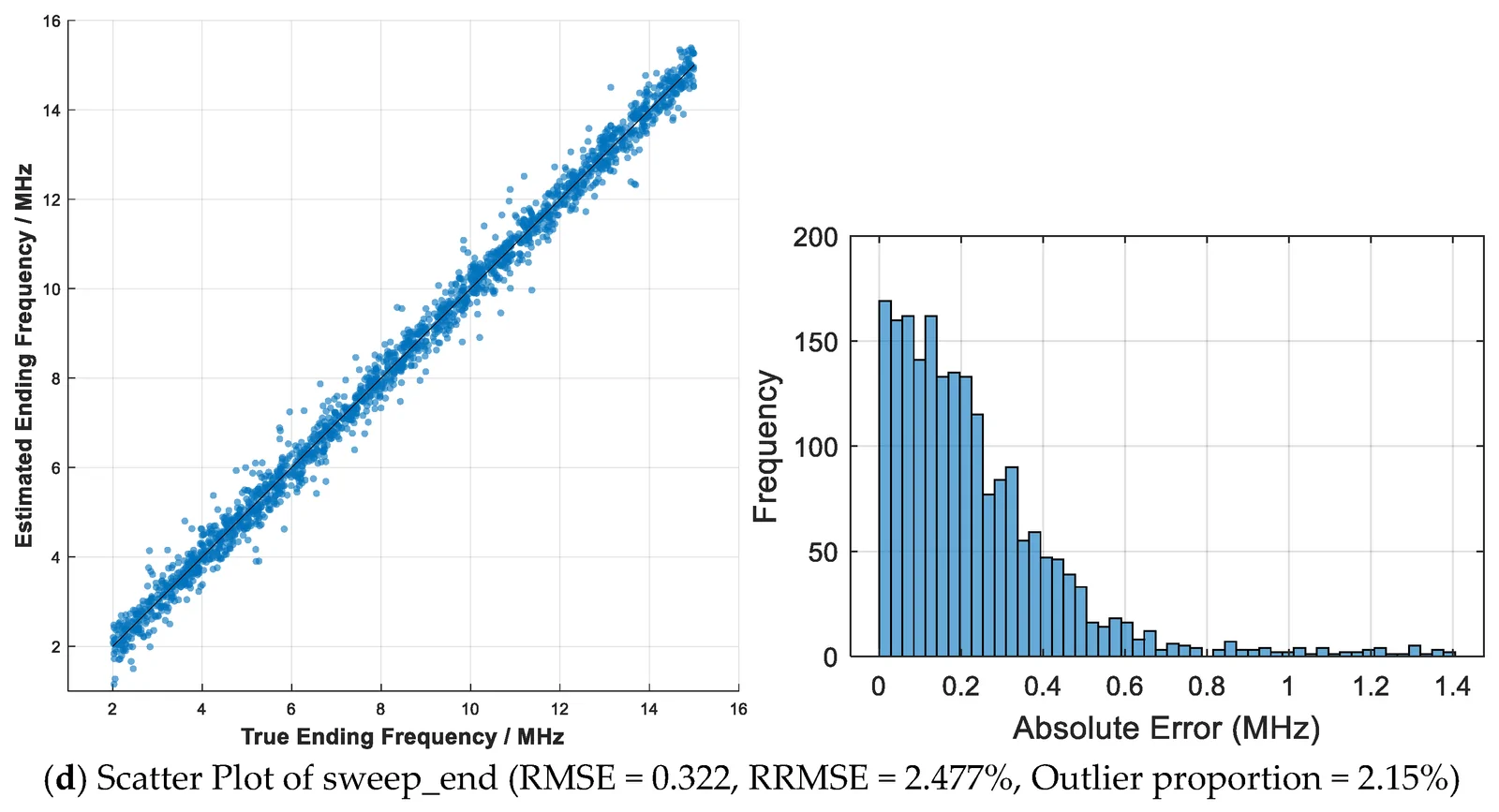
Parameter estimation scatter plot.

Figure 5 and Figure 6 present the output results of the model on Test Set 1. Specifically, Figure 5 shows the confusion matrix for the classification results. In the confusion matrix, the recognition accuracies for PI, CI, and CWI all exceed 96.1%, indicating that the model can accurately identify and effectively distinguish interference signals with distinct time-frequency structural characteristics. The analysis also reveals that the lower classification accuracy primarily stems from misclassification between WNI and the Clean background, with a misclassification rate of approximately 10%. This is because, compared with other interference types, WNI has a broader spectrum and is more susceptible to noise under the same low INR conditions.

Figure 6 shows the parameter estimation results, including a scatter plot of parameter distribution and a histogram of error distribution. In the scatter plot, the horizontal axis represents the true values, and the vertical axis represents the estimated values. The solid black line represents the ideal estimation outcome, i.e., the linear relationship between the true values and the estimated values, which is referred to as the reference line. The closer the actual estimation results are to this reference line, the higher the estimation accuracy. In the histogram of error distribution, the horizontal axis represents the absolute error, and the vertical axis represents the frequency.

When evaluating the parameter estimation performance in Figure 6, the Root Mean Square Error (RMSE) and the Relative Root Mean Square Error (Relative RMSE) are selected as the overall parameter error metrics, and their expressions are respectively

(12) $$RMSE=\sqrt{\frac{1}{N}\sum_{i=1}^{N}{\left(y_{i}-{\hat{y}}_{i}\right)}^{2}},$$

(13) $$RRMSE=\frac{\sqrt{\frac{1}{N}\sum_{i=1}^{N}{\left(y_{i}-{\hat{y}}_{i}\right)}^{2}}}{max(y)-min(y)},$$

where $y_{i}$ is the true parameter value, ${\hat{y}}_{i}$ is the estimated parameter value, N is the number of samples, and max(y)−min(y) is the range of variation of the true values. The proportion of outliers lying outside the boundaries of ±2×RMSE is adopted as a measure of the dispersion of the parameter error.

In Figure 6, the parameter types are arranged in the following order: [tone_freq, sweep_start, sweep_period, sweep_end]. These four parameters can be categorized into two classes: frequency parameters and time parameters.

For the frequency-related parameters of the signals—including the center frequency of single-tone interference, the starting frequency of sweep interference, and the ending frequency of sweep interference—the model achieved relatively precise overall estimation on Test Set 1, with RMSE values ranging from 0.26 to 0.33 MHz and RRMSE values ranging from 1.9% to 2.5%. Combined with the scatter plot, the error histogram, and the outlier proportion, it can be observed that the majority of the scatter points are concentrated near the reference line, with only a small number of outliers (approximately 1.9–2.2%), which arise from the significant estimation errors introduced by blurred signal characteristics under low INR conditions. However, these outliers do not obscure the model’s favorable performance in most scenarios. This result confirms the inherent advantage of convolutional layers in extracting localized and translation-invariant features from time-frequency representations, as such features maintain a robust mapping relationship with frequency information.

For the time-related parameter of the signals—the sweep period of chirp interference—the outlier proportion is 1.85%, which is similarly low compared with other parameters. However, its RMSE is 1.069 μs and its RRMSE is 5.345%, both significantly higher than those of the frequency-related parameters, indicating poorer estimation accuracy. This is because estimating the sweep period requires identifying and measuring the time interval between periodic structural events, which depends on integrating global context across cycles. This result highlights the difficulty of the CNN’s local filtering-pooling hierarchical structure in establishing such long-range temporal dependencies, leading to significantly lower estimation accuracy for time-scale parameters compared to frequency-domain parameters. Furthermore, under low INR conditions, the already-challenging long-range temporal dependencies become even more difficult to capture, which exacerbates the difficulty of period estimation.

To further evaluate the model’s generalization capability under more challenging conditions, additional challenging experimental tests were conducted on Test Set 3. The results show that the model achieves an overall classification accuracy of 87.6%, with RMSE values of 0.312 MHz for CWI center frequency estimation, 0.389 MHz for CI starting frequency estimation, 0.394 MHz for CI ending frequency estimation, and 1.893 μs for CI sweep period estimation, all exhibiting a degradation compared with the performance under the baseline channel.

This performance degradation can be attributed to two types of distortion effects introduced by time-varying/fading channels. First, the frequency-selective fading caused by multipath effects distorts the frequency-domain distribution of interference energy in the spectrogram, while intersymbol interference resulting from multipath delay spread further reduces the temporal resolution of the spectrogram, blurring the time-frequency boundaries of the interference. This effect is particularly pronounced for the estimation of the CI sweep period, which relies on global temporal structure. Second, the Doppler shift and its associated spectral broadening effect disrupt the time-frequency structural consistency of the interference signal, causing frequency shift and broadening of the originally linear chirp trajectory under the baseline channel, which directly affects the estimation accuracy of frequency-related parameters (CWI center frequency, CI start and stop frequencies). Nevertheless, the model still maintains usable classification and estimation performance under unseen time-varying/fading conditions, verifying that the learned time-frequency features possess a certain degree of cross-channel generalization capability.

To validate the robustness of the results, four additional independent repeated training experiments were conducted in this section. The results show that the outcomes of the multiple experiments are highly similar to those of the single experiment described above, confirming the reliable stability of the aforementioned conclusions.

#### 4.2.2. Ablation Study Design and Robustness Analysis

The dual-branch CNN model presented in this paper is a specific network that shares two convolutional layers (referred to in this subsection as the Dual-Branch-2 network). To validate the model performance, an architectural ablation study is conducted, in which network models sharing 0, 1, 3, and 4 layers are designed, denoted as the Dual-Branch-0, Dual-Branch-1, Dual-Branch-3, and Dual-Branch-4 networks, respectively. Among these, the Dual-Branch-0 network is highly similar to the serial architecture that performs classification first and then estimation, with the only difference being that the Dual-Branch-0 network executes multi-task parallel outputs. In the following, the classification and parameter estimation accuracy, as well as the robustness of each model, are tested on Test Set 2, and the results under five INR levels are compared. In view of the randomness inherent in model training, we performed five independent repeated training runs for each model and tested the model from each run. The final results were obtained by averaging the outcomes of the five tests, and the corresponding standard deviations were recorded.

**Figure 7:**
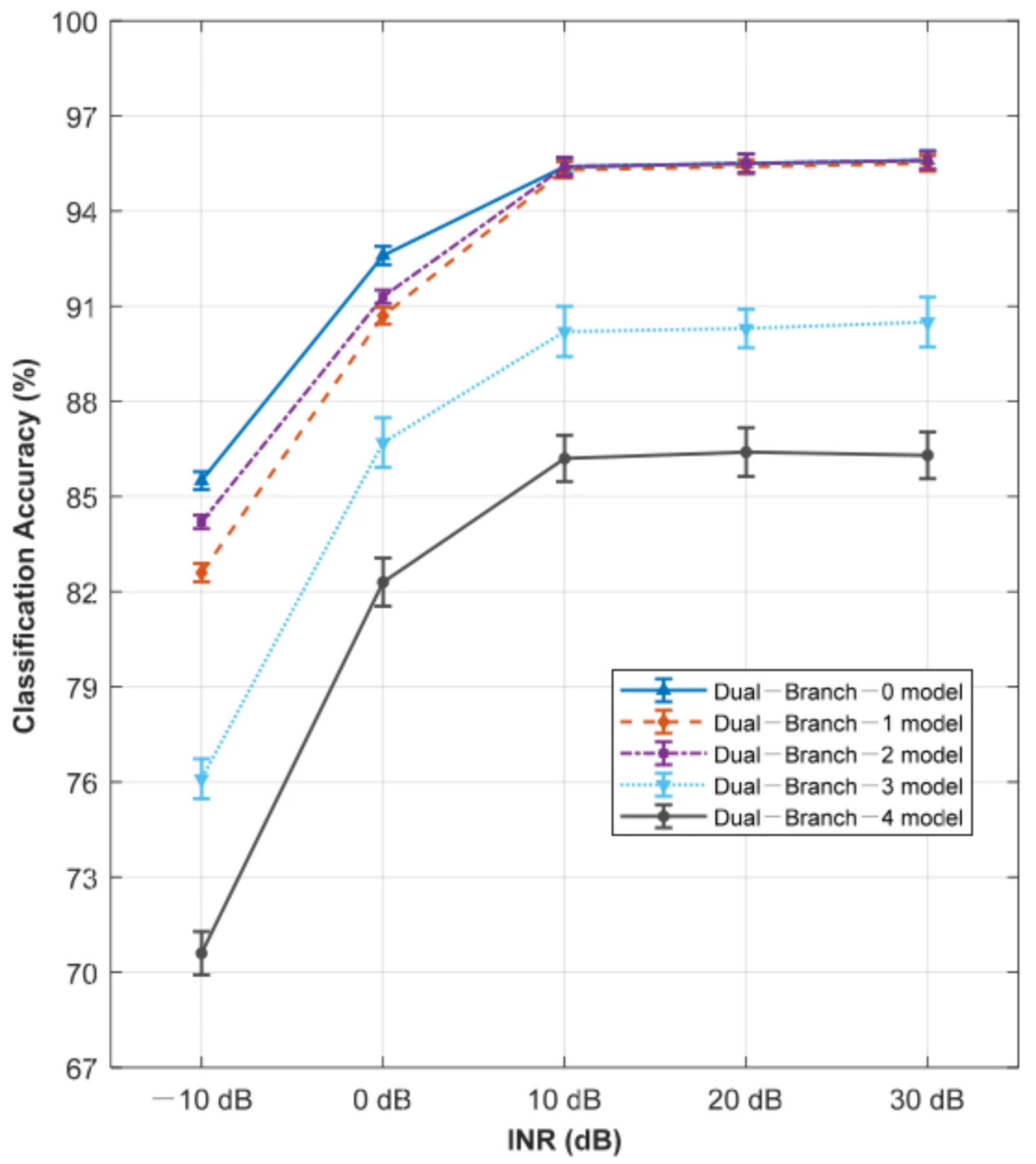
Comparison of classification accuracy for each model under different INR levels (vertical tick marks indicate ±1 standard deviation).

Table 3 presents the overall classification accuracy of the Dual-Branch-2 network model under different INR levels. Figure 7 shows a line graph depicting the variation in overall classification accuracy for the five network architectures across different INR levels. It should be noted that in Figure 7, error bars (mean ± standard deviation) are indicated for each model. As can be seen from the figure, the overall standard deviations of all error bars are relatively small, ranging between 0.2% and 0.8%, indicating good consistency across the model training runs. Consequently, the conclusions drawn from the subsequent analyses based on the mean values are highly reliable. The results in the figure indicate that the classification accuracy for all models is lowest at an INR of −10 dB. Within the INR range from −10 dB to 10 dB, the classification accuracy of all models progressively increases as the INR rises. The accuracy tends to stabilize once the INR reaches 10 dB or higher. The underlying reason is that at lower INR levels, the energy of the interference signal is relatively weak, and its characteristics are easily masked by noise, making it difficult for the models to extract discriminative features effectively, thus limiting classification performance. As the INR increases, the salience of interference features in the received signal is enhanced, allowing the models to extract more stable and distinguishable patterns more easily, thereby improving classification accuracy. Once the interference feature strength exceeds a certain threshold, the models can maintain stable recognition performance. Specifically, the Dual-Branch-0 model performs optimally under most INR conditions, achieving an accuracy of 85.5% at −10 dB and gradually reaching 95.6% as the INR increases to 30 dB. The Dual-Branch-1 and Dual-Branch-2 models also perform well, with their accuracy consistently above 82%, and their performance nearly matches that of the Dual-Branch-0 model at high INR levels (10–30 dB). This suggests that when interference features are sufficiently prominent, several network architectures can fully utilize their discrimination capabilities. In contrast, the classification accuracy of the Dual-Branch-3 and Dual-Branch-4 models at low INR (−10 dB) falls below 77% and 71%, respectively. Although their performance improves with increasing INR, it remains lower than that of the first three models across the entire INR range, with the performance gap being most significant in the −10 dB to 10 dB interval. Regarding robustness, distinct differences exist among the models: the Dual-Branch-0 model maintains the leading performance with the least fluctuation across the entire INR range; the Dual-Branch-2 model performs slightly worse at low INR, followed closely by the Dual-Branch-1 model, but both rapidly approach optimal performance as INR increases; the Dual-Branch-3 and Dual-Branch-4 models exhibit overall lower accuracy and greater variation with changing INR, indicating their relatively weaker robustness against interference.

These differences in performance and robustness primarily stem from the design of feature-sharing depth in different dual-branch architectures. The Dual-Branch-0 model, with no shared layers, avoids potential inter-task conflicts and demonstrates superior performance across all INR levels. The Dual-Branch-2 model achieves a balance between the benefits of feature reuse and the impact of task conflict through shallow-layer sharing. Although sharing low-level features enhances data utilization efficiency and generalization capability, a certain level of conflict exists between the classification and regression tasks, where the cost slightly outweighs the benefit. The Dual-Branch-1 model follows a similar principle but achieves a slightly less optimal balance than the Dual-Branch-2 model. These two models exhibit slightly weaker feature extraction capability at low INR. However, as interference features strengthen, their network capacity is sufficient to support high-accuracy classification. Conversely, the Dual-Branch-3 and Dual-Branch-4 models, likely due to an excessive number of shared layers, cause high-level feature extraction to be constrained by multi-task gradient conflicts. This is particularly detrimental under low INR conditions where feature discriminability is already limited, thereby affecting classification stability and final accuracy.

Table 4 presents the Root Mean Square Error (RMSE) for each parameter of the Dual-Branch-2 network under different Interference-to-Noise Ratio (INR) levels. It should be noted that the error for the ending frequency of the Chirp Interference is not included in this evaluation. Instead, the sweep rate parameter k which is not a direct output of the model, is newly introduced. This approach is primarily based on the relationship $k=\frac{f_{e}-f_{0}}{T}$, where the sweep starting frequency $f_{0}$, ending frequency $f_{e}$, and sweep period T constitute low-level features that are more readily learned by the model. Furthermore, the RMSE of the k value directly influences the suppression performance of precise anti-interference measures, making it an essential parameter for achieving accurate interference mitigation.

**Figure 8:**
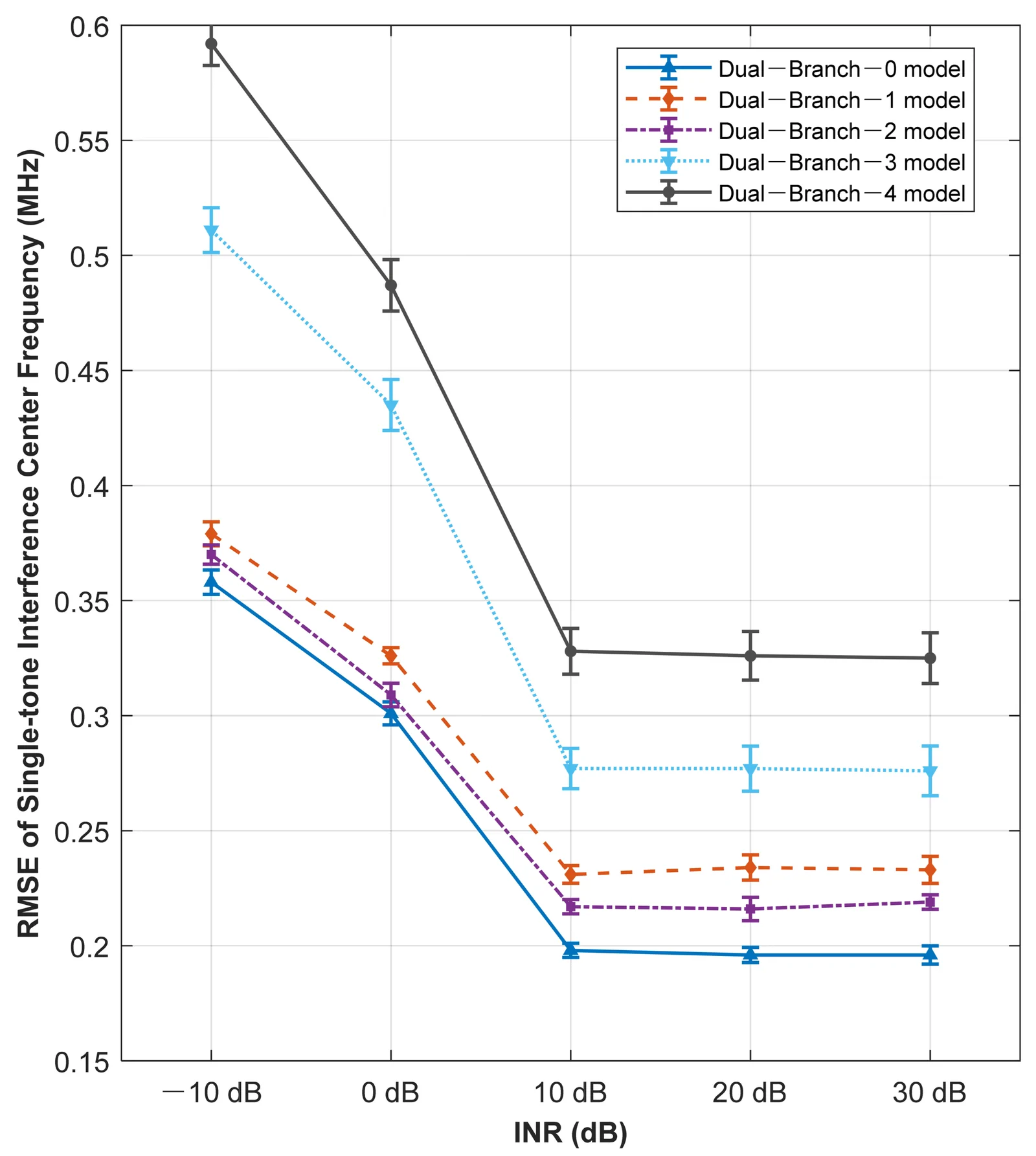
Comparison of center frequency RMSE for each model under different INR levels (vertical tick marks indicate ±1 standard deviation).

Figure 8 presents the RMSE of the center frequency of single-tone interference for the five networks under different INR levels, with error bars annotated in the same manner as in Figure 7. The overall standard deviations of all error bars are relatively small, ranging between 0.003 MHz and 0.012 MHz, indicating that the conclusions drawn from the subsequent analyses based on the mean values are highly reliable.The results in the figure indicate that the RMSE for all models is highest at an INR of −10 dB. As the INR increases from −10 dB to 30 dB, the estimation error for all models shows a significant downward trend and gradually stabilizes after the INR reaches 10 dB. This phenomenon aligns with the pattern observed for classification accuracy, stemming from the fact that interference features are more ambiguous at low INR, limiting parameter estimation performance. However, as the INR improves, the interference features become more prominent, enabling the models to extract more stable and finer-grained signal characteristics, thereby enhancing task performance.

Regarding the specific performance of each model in the parameter estimation task, the overall trend is highly similar to the pattern observed in the classification task. However, a noteworthy subtle distinction exists: under high INR conditions, the Dual-Branch-0, Dual-Branch-1, and Dual-Branch-2 models exhibit a clear hierarchy in parameter estimation accuracy. Specifically, the performance of the Dual-Branch-0 model consistently remains slightly better than that of Dual-Branch-2, which in turn is slightly better than Dual-Branch-1. This differs from the nearly identical performance observed among the three in the classification task. The reason for this discrepancy lies in the fact that classification primarily relies on extracting discriminative overall patterns from the signal. When the INR rises to a high level, the interference features themselves become extremely prominent, and all model types can relatively easily capture the decisive features needed for classification, causing the classification accuracy of all models to enter a high-level saturation zone. In contrast, parameter estimation is highly sensitive to local details and continuous variations. Even under high INR, there remains room for more precise characterization and quantification of subtle signal features. The higher accuracy of the Dual-Branch-0 model confirms this. In the Dual-Branch-2 model, a certain degree of task conflict exists between the classification and regression tasks. The benefits of feature reuse gained from sharing shallow layers and the targeted optimization of independent high-level branches cannot completely eliminate this impact. This results in the Dual-Branch-2 model’s accuracy being slightly weaker than that of the Dual-Branch-0 model under high INR, indicating that the model’s parameter estimation accuracy has not yet entered a saturation zone. The Dual-Branch-1 model shares this characteristic. The errors for other regression parameters across different networks and INR levels, as presented in the table, exhibit similar properties to those of the center frequency error and will not be listed individually.

#### 4.2.3. Comparative Experiment

This section proceeds to compare the regression parameter errors of the dual-branch CNN with those of conventional methods in order to analyze the advantages of deep learning. Regarding the classification task, the advantages of deep learning have already been established in Reference [16], and the core contribution of the proposed model lies in the simultaneous output of classification and regression tasks; therefore, the comparative value of the classification task is limited and will not be further elaborated. In contrast, parameter estimation imposes more stringent requirements on the fidelity of time-frequency details in signals, and comparative experiments can better demonstrate the structural advantages of deep learning in regression tasks.

Table 5 presents a comparison of the RRMSE of CWI center frequency between the dual-branch CNN and the existing FFT interpolation frequency estimation method [22]. The results show that while the traditional FFT interpolation method can achieve measurement accuracy (RRMSE < 0.0007%) close to the theoretical limit under high INR (≥10 dB) conditions, its performance heavily relies on ideal strong interference scenarios. In medium-to-low INR environments, the method’s performance degrades sharply or even fails. In contrast, the dual-branch CNN architecture overcomes the dependency on high INR inherent in traditional methods: even in scenarios with INR as low as −10 dB, it maintains a usable estimation accuracy of 2.64%, enabling early interference detection and advancing warning capabilities. As the INR increases, its performance rapidly converges to a stable value, demonstrating favorable robustness and environmental adaptability.

Table 6 presents a quantitative comparison between the dual-branch CNN and the existing time-frequency ridge extraction method [23] for the sweep interference parameter estimation task. The results indicate that the robustness of the time-frequency ridge extraction method exhibits characteristics similar to those of the traditional FFT interpolation approach: it achieves effective estimation under high INR conditions (at INR = 30 dB, the RRMSEs of the three parameters are all within 6%), but suffers from severe performance degradation in low-INR scenarios (when INR decreases from 30 dB to −10 dB, the RRMSEs of the three parameters increase by 12.79% to 24.71%). The underlying reason for this phenomenon is that the time-frequency ridge extraction method heavily relies on manually designed time-frequency transform parameters and explicit ridge-tracking strategies; when INR decreases, energy diffusion in the time-frequency images causes ridge breakage, hopping, or erroneous tracking, leading to a collapse in estimation accuracy. In contrast, the dual-branch CNN, through its end-to-end learning paradigm, does not depend on manual parameter tuning or explicit search rules, and is capable of maintaining stable and continuous parameter estimation performance under any INR condition (at INR = 30 dB, the RRMSEs of the three parameters are all within 4%; when INR decreases from 30 dB to −10 dB, the RRMSEs of the three parameters increase by only 0.65% to 2.24%).

In the preceding sections, we have presented ablation analyses for the multi-task deep learning framework and comparative experiments against traditional algorithms. It should be noted, however, that the deep learning comparisons conducted earlier did not include the complex single-task networks reported in [16,17,18,19,20]. The reason is that the parameter scales of those networks differ substantially from the architecture adopted in this work; a direct comparison would inevitably confound the benefits of multi-task collaboration with the advantages of network capacity, thus failing to answer the mechanism question of shared layer depth that this paper aims to investigate. Based on this consideration, we construct single-task classification and single-task regression networks that differ from the dual-branch network only in the number of shared layers as baselines. To comprehensively analyze the advantages of the proposed model, we compare these baselines from multiple aspects, including accuracy, real-time performance, and lightweight design, and place this baseline comparison in the practicality analysis of Section 4.2.4.

#### 4.2.4. Practicality Analysis

Table 7 presents a comprehensive comparison of various metrics for each model, where the RMSE of the CWI center frequency is adopted as the representative metric for parameter estimation RMSE. Three baselines are additionally included: single-task classification, single-task regression, and a single-task serial architecture. Analysis of the classification accuracy and the CWI center frequency RMSE reveals that the Dual-Branch-2 model is only slightly inferior to the Dual-Branch-0 model and the single-task serial architecture, with classification accuracy being approximately 0.38% and 0.32% lower, respectively, while the CWI center frequency RMSE is about 4.3% and 4.7% higher, respectively.

The following analyzes the lightweightness and real-time performance of the model. Regarding lightweightness, since battlefield environments are highly dynamic and demand rapid model response, the model is generally deployed onboard. On onboard equipment with limited computational power, model lightweightness becomes extremely critical. A qualitative analysis of model lightweightness is first conducted. The model adopts a relatively concise CNN architecture, which avoids invoking excessive modules while preserving accuracy—this constitutes a key advantage of the model. A detailed analysis reveals that the network parameter count decreases with increasing shared depth: the Dual-Branch-3/4 networks share the most layers, thus possessing the fewest parameters and being the most lightweight; the Dual-Branch-0 architecture, due to its fully independent branches, has the largest parameter count, while the Dual-Branch-1/2 architectures fall in between. In the quantitative evaluation, the peak inference memory usage of the Dual-Branch-2 structure is approximately 240 MB, which is about 26.2% lower than that of the Dual-Branch-0 model.

In terms of real-time performance, a qualitative analysis indicates that the computational parallelism of the model directly affects the inference speed: the Dual-Branch-3/4 networks, with highly shared computation paths, achieve the highest parallelism and the shortest inference time; the Dual-Branch-0 network, with independent branches, has low parallelism and the slowest speed, and the serial network with similar accuracy is a sequential architecture, which incurs multiplied inference time and is prone to cascading errors caused by misclassification; the inference speeds of the Dual-Branch-1/2 networks fall in between. In the quantitative evaluation, the single-inference time of the Dual-Branch-2 structure is 14 ms, which is approximately 14.3% faster than that of the Dual-Branch-0 model and 37.2% faster than that of the single-task serial architecture. In summary, when all metrics are comprehensively considered, the Dual-Branch-2 structure outperforms the other model architectures.

Under conventional airborne deployment conditions, embedded platforms such as the Jetson Orin Nano/Xavier NX are equipped with 4–8 GB of shared memory, and the GNSS receiver anti-interference processing cycle is typically around 20–50 ms, which fully satisfies the memory and inference requirements of the Dual-Branch-2 model.

The above experiments demonstrate that the dual-branch CNN model, by sharing two convolutional layers, identifies the optimal point where the feature reuse benefit curve and the task interference cost curve intersect in this scenario, thereby achieving the best overall balance among accuracy, lightweightness, and real-time performance.

## 5. Discussion

The dual-branch CNN model proposed in this paper exhibits favorable performance and robustness in jamming classification and parameter estimation tasks, and several characteristics of the experimental results warrant in-depth discussion.

First, the confusion matrix analysis reveals a phenomenon worthy of attention: a relatively high mutual misclassification rate exists between WNI and the no-jam background, and this phenomenon is primarily concentrated in the low INR regime. From the signal nature perspective, the spectral characteristics of WNI are highly similar to those of thermal noise—both manifest as a relatively uniform power distribution in the frequency domain, with the only distinction being that the power spectral density of the interference is significantly higher than the noise floor. Under low INR conditions, this difference is masked by noise, making it difficult for the model to extract effective discriminative features. This finding exposes an inherent limitation of deep learning methods based on time-frequency images: when the statistical properties of the jamming signal converge with those of the background noise, pattern recognition that solely relies on the time-frequency energy distribution will face fundamentally ambiguous boundaries. In the future, higher-order statistics (e.g., amplitude distribution and cyclostationary features) can be introduced as auxiliary input channels to enhance the model’s discriminative capability under low INR conditions.

Second, the disparity in accuracy between frequency-domain parameters and temporal parameters in the parameter estimation task offers significant architectural insights. Experimental results demonstrate that the CNN estimates the CWI center frequency and the chirp start/end frequencies with much greater precision than the chirp sweep period. This is not fortuitous but is determined by the intrinsic inductive bias of the CNN architecture. The core operations of CNNs—local convolution and pooling—are inherently suited to extracting local spectral patterns with translation invariance, which highly aligns with the physical properties of frequency parameters. In contrast, the chirp sweep period, as a global temporal structure spanning multiple pulse cycles, requires the network to establish long-range temporal dependencies for effective estimation, which is precisely a weakness of standard CNNs. Although the shared shallow convolutional layers can capture certain local temporal segments, the lack of global temporal modeling capabilities, such as recurrent structures or attention mechanisms, fundamentally constrains the upper bound of period estimation accuracy. This limitation suggests that a hybrid architecture design combining CNNs with temporal modeling modules, or the introduction of explicit physical constraints (e.g., periodicity prior regularization), may serve as effective pathways to further improve the estimation accuracy of temporal parameters.

Furthermore, the ablation study reveals the intricate trade-off between sharing depth and task conflict in multi-task learning. The Dual-Branch-2 model achieves the optimal overall balance among accuracy, lightweightness, and real-time performance, yet its parameter estimation accuracy remains marginally inferior to that of the serial network under high INR conditions. This corroborates a core hypothesis: a certain degree of gradient conflict indeed exists between the classification and regression tasks in the high-level feature space—classification pursues maximization of inter-class discriminability, tending to amplify feature differences, whereas regression pursues continuous accuracy in parameter estimation, tending to preserve fine-grained numerical structures. The optimization directions of the two tasks for feature representation are not entirely aligned, and when the shared layers are excessively deep, such conflicts will inflict non-negligible harm on performance. However, although the serial network holds a slight advantage in accuracy, it significantly increases inference time and memory footprint due to the absence of feature reuse, and its sequential processing architecture is prone to cascading errors triggered by misclassification. By constraining the sharing depth to the shallow convolutional layers, the Dual-Branch-2 model preserves the benefit of low-level feature reuse while delegating the optimization of task-specific representations to the independent branches, thereby achieving an effective practical compromise between engineering feasibility and theoretical optimal accuracy. Furthermore, it is necessary to clarify the rationale for selecting the number of shared layers as the design dimension in the ablation study. The number of shared layers is a critical design variable in hard parameter sharing multi-task architectures, with its theoretical root lying in the fact that it determines the boundary between general feature extraction and task-specific representation. When the shared layers are too shallow, each task branch lacks effective reuse of low-level features, and independent learning may lead to overfitting. When the shared layers are too deep, the gradient update directions of the classification and regression tasks conflict in the high-level space, inducing task interference. Therefore, the number of shared layers essentially serves as a control variable regulating information flow and gradient conflict between tasks. The experimental results in this paper intuitively demonstrate the sensitivity of this design variable—significant performance variations are observed across different sharing depths, which in turn validates its appropriateness as a key subject for ablation investigation.

It is worth noting that the research orientation of this paper is fundamentally different from the mainstream directions in recent multi-task learning (MTL) studies. Current state-of-the-art MTL work predominantly focuses on algorithmic-level improvements—such as dynamic task weighting, gradient surgery, and uncertainty weighting—aiming to adaptively balance the convergence speed and optimization directions of different tasks during training. These works treat the network architecture (e.g., the number of shared layers) as a given parameter rather than as an object of study, lacking systematic exploration of its underlying mechanisms. As a result, the interpretability of architectural advantages remains weak, and in some cases may even compromise model performance. In contrast, this paper restricts its scope to static architectural design under hard parameter sharing, with the core concern being how the depth of shared layers affects the upper bound of information sharing between tasks and the degree of task conflict. To purely observe the main effect of this architectural factor, this paper, based on the principle of controlled variables, does not introduce dynamic weighting or gradient manipulation strategies. Introducing algorithmic-level interventions would make it impossible to attribute any performance variation to the sharing depth itself, thereby undermining the core conclusion of this study.

In this sense, this paper and existing algorithmic-level MTL work are orthogonal and complementary to each other: this paper answers the question of which architectural starting point is optimal, while existing algorithmic work addresses how to achieve more stable optimization given a particular starting point. Their combination represents a future direction for unlocking performance limits. However, the architectural findings of this paper possess independent guiding value—that is, regardless of the optimization strategy adopted, choosing a sharing depth near the bottom of the U-shaped curve constitutes a superior starting point. Future work may further introduce algorithmic strategies such as weighting allocation or gradient manipulation on top of the optimal sharing depth criterion revealed in this paper, in order to explore the superimposed gain from combining architectural optimality and training optimality.

Furthermore, it is worthwhile to discuss the relationship between the motivation of the proposed method and engineering practice. In current GNSS anti-jamming engineering practice, blind suppression remains the most widely adopted mainstream approach. This situation is not due to technical conservatism, but rather a pragmatic choice dictated by real-world constraints: blind suppression algorithms are well-established, computationally lightweight, and simple to implement in hardware, and have been validated over long-term operation in a large number of fielded systems, with system stability and reliability fully verified. However, precisely because of their “blind” nature, these methods are consistently unable to answer three fundamental questions: what type of interference is present? what are the key parameters? and what countermeasure strategy should be configured? This lack of information means that blind suppression can only adopt generic, conservative countermeasures. In the face of increasingly diverse jamming patterns and more complex electromagnetic environments, the performance ceiling and robustness limitations of blind suppression have become increasingly evident. Persistent issues such as residual interference, signal distortion, and phase offset have been repeatedly demonstrated in recent studies and remain fundamentally unresolved to date. It is precisely here that the core motivation of this paper lies: before the interference enters the conventional anti-jamming processing chain, deep learning is first employed to simultaneously accomplish interference type identification and key physical parameter estimation, thereby transforming unknown interference into a known entity and providing an information foundation for the precise configuration of subsequent countermeasures. It is worth emphasizing that this paper does not advocate replacing blind suppression with cognitive methods; rather, the two are complementary. Blind suppression provides cost-effective and robust protection under routine scenarios, while cognitive methods offer precise perception and differentiated response capabilities in scenarios where blind suppression performs poorly. Together, they constitute a layered anti-jamming framework tailored for complex electromagnetic environments.

From a broader perspective, the significance of this study lies not only in proposing a specific network architecture but also in validating the methodological feasibility of end-to-end multi-task learning for satellite navigation anti-jamming. In the conventional sensing–decision–action chain, jamming classification, parameter estimation, and anti-jamming measure configuration are often performed serially by mutually independent modules, with fixed interfaces and unidirectional information flow, making global optimization difficult. The dual-output architecture for jamming sensing and parameter estimation constructed in this work provides crucial front-end support for building closed-loop intelligent anti-jamming systems—when the classification and estimation results can be directly mapped to specific countermeasure parameters such as notch center frequency and chirp tracking trajectory, the closed logic loop from sensing to countermeasures becomes engineeringly realizable. It is worth noting that the proposed method still has several limitations. First, the experimental data are derived from a laboratory simulation platform, where the signal models, noise models, and receiver front-end parameter settings deviate from those in actual electromagnetic environments. Although we have validated that the model possesses a certain degree of generalization capability under time-varying/fading channel tests, the combined effects of multipath, RF non-idealities, and random variations of jamming parameters in real environments still need to be verified using measured GNSS interference data. Second, the current model input assumes a time-frequency diagram of a single interference source that has been decoupled via spatial filtering. This precondition relies on the reliability of front-end array signal processing in actual composite interference scenarios. If residual coupling remains in the front-end decoupling, the model performance may suffer unforeseen degradation, which is a critical aspect that requires attention in subsequent system integration. Finally, the interference types covered in this study are still typical cases and do not encompass all possible interference patterns. In future work, a more comprehensive training set covering a wider variety of interference types and real-world collected data should be constructed to enhance the model’s adaptability to unknown environments and interference patterns. At the system integration stage, the perception module proposed in this paper should be jointly optimized with the front-end spatial processing to suppress the impact of residual front-end decoupling errors on perception performance. Moreover, the optimal sharing depth criterion revealed in this paper could be combined with more advanced network backbones to improve the representation capability for complex time-frequency structures.

## 6. Conclusions

To address the problems of satellite navigation interference signal classification and parameter estimation, this paper proposes a multi-task learning method based on a dual-branch convolutional neural network. The network adopts a four-layer convolutional architecture, sharing the first two layers as a feature extraction backbone, upon which dedicated branches are constructed to simultaneously accomplish interference classification and parameter estimation.

The main contributions of this paper are threefold: it reveals a U-shaped relationship between the sharing depth and the classification/regression performance in hard parameter sharing multi-task architectures, demonstrating that moderate sharing achieves the optimal balance among accuracy, lightweight design, and real-time performance, while excessive sharing induces gradient conflicts and performance degradation between tasks; it provides a quantitative design criterion for the optimal sharing depth in this scenario, offering a reproducible architectural reference for resource-constrained airborne deployments; and it verifies the systematic advantages of multi-task parallel processing over the serial classify-then-estimate paradigm in terms of latency accumulation and resource occupancy.

This study demonstrates that in multi-task learning frameworks, static architectural design constitutes a dimension orthogonal to but equally important as dynamic optimization algorithms.

## Figures and Tables

**Table 1:** Model regression tasks and relevant logic.

Interference Type	Key Parameters	Adaptive Anti-Interference Measure	Measure Configuration Logic
Continuous Wave Interference	Center Frequency$\;f_{c}$	Narrowband Notch Filtering	The notch center frequency is strictly matched to$\;f_{c}$, with a bandwidth set to 100 kHz, to attenuate and eliminate the interference.
Chirp Interference	Starting Frequency$\;f_{0}$ Sweep Period T Ending Frequency$\;f_{e}$	Tracking Notch Filtering	The notch center frequency is updated in real time according to$\;f_{0}+kt$, where$\;k=\frac{f_{e}-f_{0}}{T}$. The notch period is synchronized with T to ensure full coverage of the interference frequency band throughout its sweep.

**Table 2:** Simulation parameters.

Type	Parameters
BeiDou B1C Signal [21]	Signal Power: −152 dBW Modulation Scheme: QMBOC (6, 1, 4/33) Effective Bandwidth: ~30 MHz
Interference Signal	Signal Power: −142 dBW to −102 dBW * INR: −10 dB to 30 dB CWI center frequency: −7 to 7 MHz CI starting frequency: −15 to −2 MHz CI ending frequency: 2 to 15 MHz CI sweep period: 5 to 25 μs
Background Noise	Type: Additive White Gaussian Noise (AWGN) Noise Power: −132 dBW

* INR = P_jamming − P_noise, in dB.

**Table 3:** Overall classification accuracy of the Dual-Branch-2 network model.

INR/dB	Dual-Branch CNN
−10	84.2%
0	91.3%
10	95.4%
20	95.5%
30	95.6%

**Table 4:** RMSE of each parameter for the Dual-Branch-2 model.

INR/dB	Continuous Wave Interference	Chirp Interference		
	Center Frequency Error/MHz	Starting Frequency Error/MHz	Sweep Rate Error/(MHz/μs)	Sweep Period Error/μs
−10	0.370	0.476	0.109	1.41
0	0.309	0.376	0.099	1.19
10	0.217	0.235	0.075	0.92
20	0.216	0.234	0.069	0.87
30	0.219	0.236	0.071	0.85

**Table 5:** Comparison of the RRMSE of CWI center frequency between the model and traditional methods.

INR/dB	Dual-Branch CNN	FFT Interpolation Frequency Estimation Method
−10	2.64%	
0	2.21%	
10	1.55%	0.000672%
20	1.54%	0.000684%
30	1.56%	0.000653%

**Table 6:** Comparison of RRMSEs of CI starting frequency, sweep rate, and sweep period between the model and traditional methods.

INR/dB		CI Starting Frequency	CI Sweep Rate	CI Sweep Period
−10	Dual-branch CNN	3.66%	1.87%	5.64%
	Time-frequency ridge extraction method	24.65	30.13	18.45
0	Dual-branch CNN	2.89%	1.70%	4.76%
	Time-frequency ridge extraction method	12.86	15.51	11.26
10	Dual-branch CNN	1.81%	1.28%	3.68%
	Time-frequency ridge extraction method	4.56	6.25	6.12
20	Dual-branch CNN	1.80%	1.18%	3.48%
	Time-frequency ridge extraction method	4.17	5.63	5.82
30	Dual-branch CNN	1.82%	1.22%	3.40%
	Time-frequency ridge extraction method	4.06	5.42	5.66

**Table 7:** Comprehensive comparison of various models.

Model	* Classification Accuracy (%)	* RMSE of CWI Center Frequency (MHZ)	Inference Time (ms)	Peak Inference Memory Usage (MB)
Dual-Branch-0	92.87	0.256	16.0	325
Dual-Branch-1	91.83	0.286	15.2	290
Dual-Branch-2	92.52	0.267	14.0	240
Dual-Branch-3	86.93	0.361	12.5	170
Dual-Branch-4	82.51	0.419	10.5	73
Single-task classification	92.82		11.1	82
Single-task regression		0.255	10.8	85
Single-task serial architecture	92.82	0.255	22.3	170

* The classification accuracy and CWI center frequency RMSE in the table are obtained from tests on Test Set 1. The results are presented as the mean over five independent test runs, with standard deviations all within 6%; the fluctuations are relatively small and can be neglected in the analysis.

## Data Availability

The data presented in this study are available upon request from the corresponding author.
